# Lab-on-Chip Cytometry Based on Magnetoresistive Sensors for Bacteria Detection in Milk

**DOI:** 10.3390/s140815496

**Published:** 2014-08-21

**Authors:** Ana C. Fernandes, Carla M. Duarte, Filipe A. Cardoso, Ricardo. Bexiga, Susana. Cardoso, Paulo P. Freitas

**Affiliations:** 1 INESC–MN—Instituto de Engenharia de Sistemas e Computadores–Microsistemas e Nanotecnologias and IN—Institute of Nanoscience and Nanotechnology, Rua Alves Redol 9, 1000-029 Lisbon, Portugal; E-Mails: fcardoso@inesc-mn.pt (F.A.C.); scardoso@inesc-mn.pt (S.C.); pfreitas@inesc-mn.pt (P.P.F.); 2 CIISA, Veterinary Medicine Faculty, Technical University of Lisbon, Av. da Universidade Técnica, 1300-477 Lisbon, Portugal; E-Mail: ricardobexiga@fmv.ulisboa.pt; 3 Physics Department, Instituto Superior Técnico, Av. Rovisco Pais, 1049-001 Lisbon, Portugal; 4 INL, International Iberian Nanotechnology Laboratory, Av.Mestre Jose Veiga, 4715-330 Braga, Portugal

**Keywords:** cytometer, microfluidic, magnetoresistive sensor, milk, *Streptococcus agalactiae*

## Abstract

Flow cytometers have been optimized for use in portable platforms, where cell separation, identification and counting can be achieved in a compact and modular format. This feature can be combined with magnetic detection, where magnetoresistive sensors can be integrated within microfluidic channels to detect magnetically labelled cells. This work describes a platform for in-flow detection of magnetically labelled cells with a magneto-resistive based cell cytometer. In particular, we present an example for the validation of the platform as a magnetic counter that identifies and quantifies *Streptococcus agalactiae* in milk.

## 1. Introduction

Flow cytometry is a technique that enables the measurement of morphological, biochemical and functional characteristics of microscopic particles (cells, viruses, bacteria, yeast, intracellular organelles, aerosol particles or microbeads (from 0.2 μm to 150 μm diameter) suspended in a stream of fluid. It allows the characterization and quantification of a population at high rates and with a high-throughput [1]. The multiparametric analysis of the particles can be performed by light scattering or staining the particles with fluorophores (fluorescent conjugated antibodies or absorption dyes) or quantum dots and presenting the particles, one by one, to a laser beam of a single wavelength or arc lamp [1–3].

For the past three decades, advances in sample pre-treatment, flow handling, precision technologies, synthesis of emitting particles, data handling techniques and bioinformatics have allowed the introduction of this sophisticated analytical tool into routine clinical and laboratory use in cell/molecular biology fields [4–6], formulation and biotesting of compounds [7,8], disease diagnosis [9–11], immunology [12,13], genetics [14], industrial bioprocesses [15], and environmental monitoring [2,16,17]. In addition to detection and enumeration, some flow cytometers have the ability to sort cells at high speeds based on detected signals without loss of viability or particle-specific characteristics [3].

Although conventional state-of-the-art flow cytometry systems provide rapid and reliable analytical results, and despite the considerable recent technological advances, these devices are still bulky, expensive and complex. Over the past years, the drawbacks of conventional flow cytometers have encouraged efforts to take advantage of microfabrication technologies and advanced microfluidics to achieve smaller, simpler, more innovative and low-cost instrumentation with enhanced portability for on-site measurements. This miniaturization approach has in general made use of inexpensive polymers such as polydimethylsiloxane (PDMS) [18] and detection techniques easily integrated with electronics [19], such as optical fibers [20], CCD cameras [21], diode lasers [22,23], PIN photodiodes [24], electrodes [25] and magnetoresistive sensors [26]. Approaches such as label-free electrical impedance-based ones [27,28], while quantitative and high throughput, present high sensitivity to the sample matrix, being affected by components in the sample other than the target, specifically their charges, which greatly hinders these devices' use in off-laboratory locations. This could also occur in fluorescent applications [29], due to non-specific adsorption of fluorophores or self-fluorescence of sample components [30]. Some platforms present a static detection [31] where labels complementary to the target are immobilized on sensor's surface. This approach, while sensitive and quantitative, is limited by the sensor's surface area and number of immobilized labels/targets, further requiring careful selection of sample flow rate.

Other sample focusing techniques have also been applied [19,20,22,32]. Along with detection and enumeration, high speed sorting has been an important theme in the development of microfabricated flow cytometers and for other applications [33–38]. Several strategies have been applied to this effect [3,19,39–44]. Regardless of the use of solid-state devices to reduce the volume of the whole system, these microfabricated systems require external equipment for the detection and enumeration of cells/particles [30].

**Figure 1.**
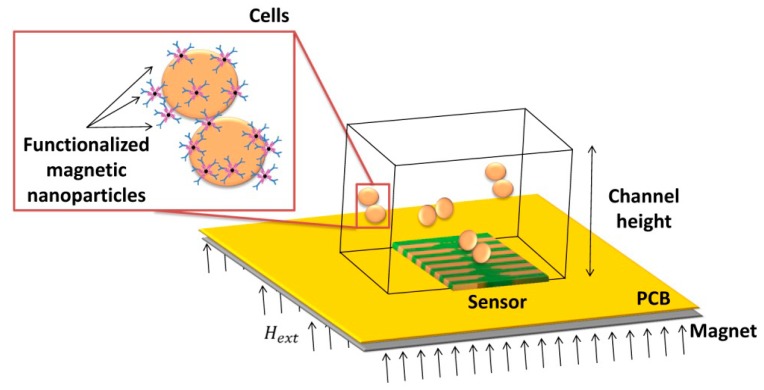
Schematics of magnetic detection device for identification and quantification of cells.

This work describes the development, characterization and application of a magnetic detection device for the identification and quantification of *Streptococcus agalactiae* in a complex matrix (milk), based on a previously developed platform [26,30], schematically depicted in Figure 1. This device comprises magnetoresistive (MR) sensors, namely spin-valve sensors (SVs), integrated with a microfluidic platform and connected to an amplification and acquisition setup. The sensors have excellent spatial resolution (on the micrometer range) and are sensitive to the magnetic field created by magnetized beads flowing in microchannels above the sensors. The detection scheme used in this platform relies on the MR sensor's sensitivity to count individual cells in flow (contrary to other approaches [45]) while providing information on nanoparticles' magnetization direction along the flow process. Therefore, no additional cell culture is needed. In addition, this platform is compatible with complex matrixes without the need of intricate sample pre-processing, while using a detection principle (magnetic) non-existent in Nature (thus greatly reducing biological background noise and false positives). The use of magnetoresistive sensors also simplifies connection with electrical equipment while still allowing coupling with other detection techniques (e.g., fluorescence or a laser-irradiated magnetic sead system (LIMBS)) if needed. This work, unlike other platforms [46–48] provides a simple approach for single cell detection, without the need for cell guiding mechanisms of hydrodynamic focusing approaches, targeting at a broader area of application—such as bacterial detection in food and water samples. To be competitive to other bulk cytometers, however, this approach should be able to accept large volumes of samples, operating with high flowrates (cm/s at least), and using multiple channels in a parallel sampling architecture.

## 2. Sensor Design and Detection Scheme

**Figure 2.**
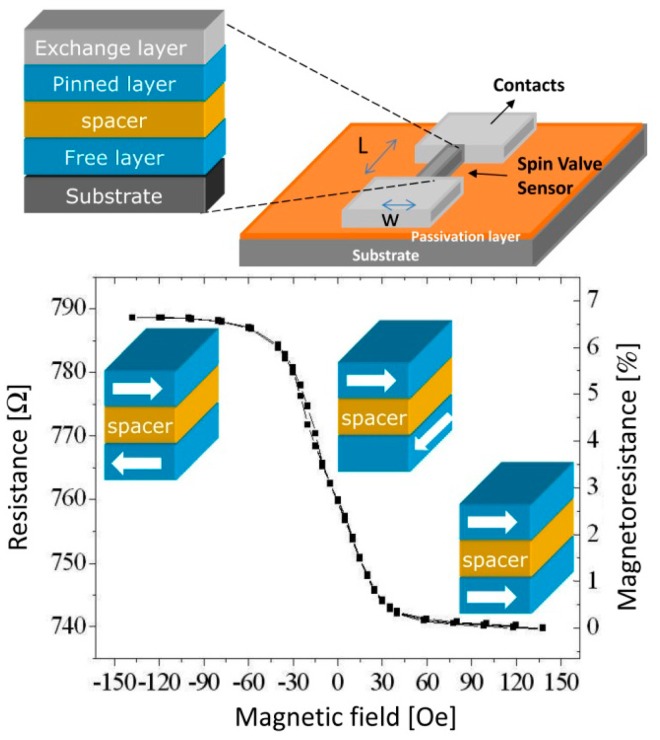
Resistance *vs*. magnetic field transfer curve of a linear spin-valve at a given sense current.

Spin-valves (SV) are magnetoresistive sensors [49,50] composed of a non-magnetic (NM) metal between two layers of ferromagnetic (FM) metals, one of which (the pinned layer) has its magnetization fixed by an adjacent antiferromagnetic layer, while the other (the free layer) is free to rotate (Figure 2). Under an external magnetic field, it is possible to switch the relative magnetic orientations of the FM layers from parallel to antiparallel, therefore changing the sensor resistance, linearly with the cosine of the relative angle between the pinned layer and the free layers (θ_p_ − θ_f_), according to Equation (1), where ΔV is the variation in potential obtained at electric current I due to sensor's resistance change. Here, MR is the sensor magnetoresistance ratio (Equation (2)) and cos (θ_p_ − θ_f_) is averaged over the active area of the sensor (between contacts):

(1) $$\Delta V=-\frac{1}{2}\left(\frac{R_{AP}-R_{P}}{R_{P}}\right)\times R_{▪}\times \frac{l}{w}\times I\times \langle \text{cos}\left(\theta_{\text{p}}-\theta_{\text{f}}\right)\rangle$$

(2) $$MR=\left(\frac{R_{AP}-R_{P}}{R_{P}}\right)$$

where, *R*_▪_ is the square resistance of the sensor, *l* the length of the sensor, *w* the width of the sensor, *I* the current applied to the sensor, *R_AP_* the resistance of the sensor when θ_p_ − θ_f_ = π and *R_P_* the resistance of the sensor when θ_p_ − θ_f_ = 0.

To achieve a linear behavior (2), the free and pinned layer easy axes should be orthogonal. The linearization can be obtained by inducing an orthogonal magnetization direction between pinned and free layers during deposition or by patterning the SV with a large aspect ratio so that orthogonal magnetizations are facilitated by the demagnetizing fields [26,30,51].

The dimensions of the SV sensors are optimized taking into account final sensor application. In biological applications, the detection targets have sizes ranging between few nm (molecules such as DNA, RNA and various proteins) to tens of μm (cells can vary in size from 1 μm, like the target cell described in this paper, to 100 μm, size of a big plant eukaryotic cell). Detection is performed through magnetic labeling of these biological targets with nano- or micrometer superparamagnetic particles, which under a magnetic field acquire a magnetic moment. This creates a fringe field that can be detected by the sensor, through a change in its resistance. Using Equation (1), if one considers a coherent magnetization model for the free layer rotation, then:

(3) $$\langle \text{cos}\left(\theta_{\text{p}}-\theta_{\text{f}}\right)\rangle =\frac{\left(H_{\text{ext}}+H_{\text{bias}}+H_{\text{coupling}}\right)}{H_{k}+H_{\text{demag}}}$$

where *H_ext_* is the external field, *H_bias_* is the bias field used to center the SV transfer curve, *H_coupling_* is the sum of the ferromagnetic Néel coupling between the free and pinned layers, *H_k_* is the free layer anisotropy field and *H_demag_* is the demagnetizing field.

Therefore the sensor output (Equation (1)) can be written as Equation (4) below:

(4) $$\Delta V=-S\times R_{P}\times I\times \left(H_{\text{ext}}+H_{\text{bias}}+H_{\text{coupling}}\right)$$

where $S=\frac{MR}{2\left(H_{k}+H_{\text{demag}}\right)}$ is the sensitivity of the sensor, $R_{P}=R_{▪}\times \frac{l}{w}$ is the resistance of the sensor when θ_p_ − θ_f_ = 0.

Here, *H_ext_* represents the external magnetic field, averaged over the sensor area. In our case this is the fringe field created by the magnetic labels [30,52,53].

**Figure 3.**
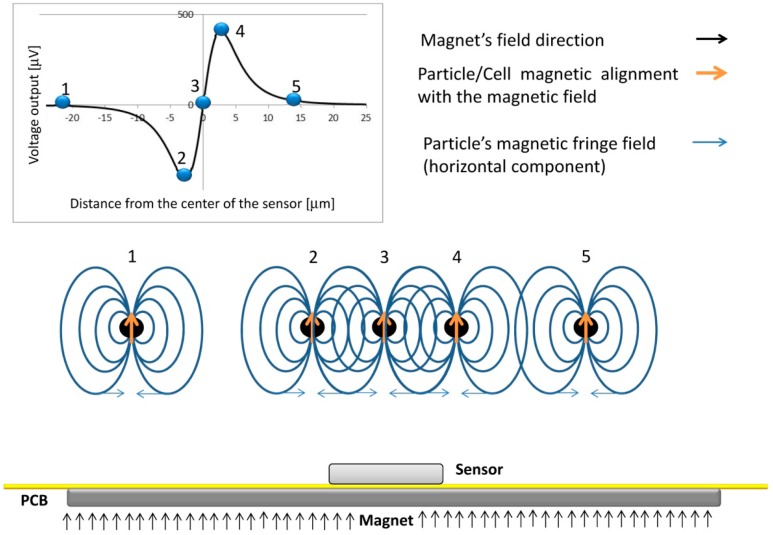
Schematics of MR sensor detection of magnetically labeled targets flowing above the sensor, from the left (position 1) to the right (position 5).

The dynamical detection approach employed in this work involves the application of a magnetic field perpendicular to the sensors in order to magnetize the beads labeling the cells, with minimum impact on the in-plane sensing direction of the sensor (as will be described in Section 3.4). The dynamic detection mechanism is illustrated in Figure 3, where a vertically magnetized particle is injected through a microchannel and generates a variable field over the sensor. In position 1, because the large distance to the sensor, the fringe field produced by the particles is neglegible. As the particle approaches the sensor, the free layer will sense the right-side component of the particles fringe field, which changes the sensor resistance (position 2). When the particle is in the center of the sensor, the average fringe field of the particle is equal to zero vanishing the signal (position 3). Finally, as the particle passes the sensor (position 4), the free layer magnetization is affected by an opposite opposite fringe field component when compared to position 2. When the cells go away, the signal goes back to zero since no fringe field is sensed (position 5). As a result, a bipolar peak is the signature of the passage of a perpendicularly magnetized particle over the SV sensor.

In a dynamical approach, sample acquisition velocity depends on the electronics, thus allowing a high throughput and direct number of cells to number of signals relation. A dynamical acquisition requires magnetic labels with a high magnetic moment under an applied external magnetic field in order to obtain a large detectable fringe field, significantly larger than the noise background level. However, label selection needs to be carefully done, as these should possess a non-remanent moment in order to avoid particle clustering during the labeling process of the cells, which can result in cell clustering originating an underestimation on the cell counting. A reduced label size is also important in order to avoid the detection of isolated particles [30,51].

## 3. Experimental Methods

One general differentiation of the Streptococci is the Lancefield groups based on serological grouping determined by the antigen C-substance that is a group-specific cell wall polysaccharide. *Streptococcus agalactiae* belongs to Lancefield Group B. Before using the selected rabbit polyclonal antibody (pAb) anti-Lancefield Group B Streptococci (8435-2000 AbDSerotec) against *Streptococcus agalactiae* cells, we have done several western blotting (WB) trials and ELISA tests for specificity and sensibility confirmation.

As known, western blotting identifies with specific antibodies proteins that have been separated from one another according to their size by gel electrophoresis. The blot is a membrane, almost always of nitrocellulose or polyvinylidene fluoride (PVDF). In our case, we have used hydrophobic PVDF membranes because it exhibits better binding efficiency of blotted proteins and have high sensitivity. Then, the gel is placed next to the membrane and application of an electrical current induces the proteins in the gel to move to the membrane where they adhere. The membrane is then a replica of the gel's protein pattern, and is subsequently stained with an antibody.

The proteins used in our WB trials were cell wall proteins of bovine field isolates of Streptococci species (agalactiae (Lancefield Group B), uberis (ungroupable) and dysgalactiae (Lancefield Group C)) and also from bacteria standards (as CECT 183 for *Streptococcus agalactiae* and CECT 994 for *Streptococcus uberis*). Results proved specificity of this pAb at 2 μg/mL concentration for 3 h of incubation at RT), to *Streptococcus agalactiae* cell wall proteins. Blots were probed with 0.2 μg/mL of goat anti-rabbit antibody conjugated to horseradish peroxidase (HRP) (STAR 124P, AbDSerotec) after one incubation hour at RT. Consequently, we have obtained stained immunogenic proteins of molecular weight higher than 100 kDa (and also two distinct immunogenic proteins around 30 and 41 kDa of molecular weight belonging to *Streptococcus uberis*).

The enzyme-linked immune sorbent assay (ELISA) is a commonly used technique to detect antibodies or antigens in samples using the specific reaction of antibodies to their antigens [54]. For pAb sensibility quantification, we have performed ELISA tests. We have used standard and field isolates of bacterial cells suspensions (agalactiae, uberis and dysgalactiae) and once more, specificity was evidenced and sensitivity obtained: the minimal pAb concentration of 1 μg/mL still identifies 10^3^ CFU/μL of *Streptococcus agalactiae* cells (and also 10^3^ CFU/μL of *Streptococcus uberis*).

**Figure 4.**
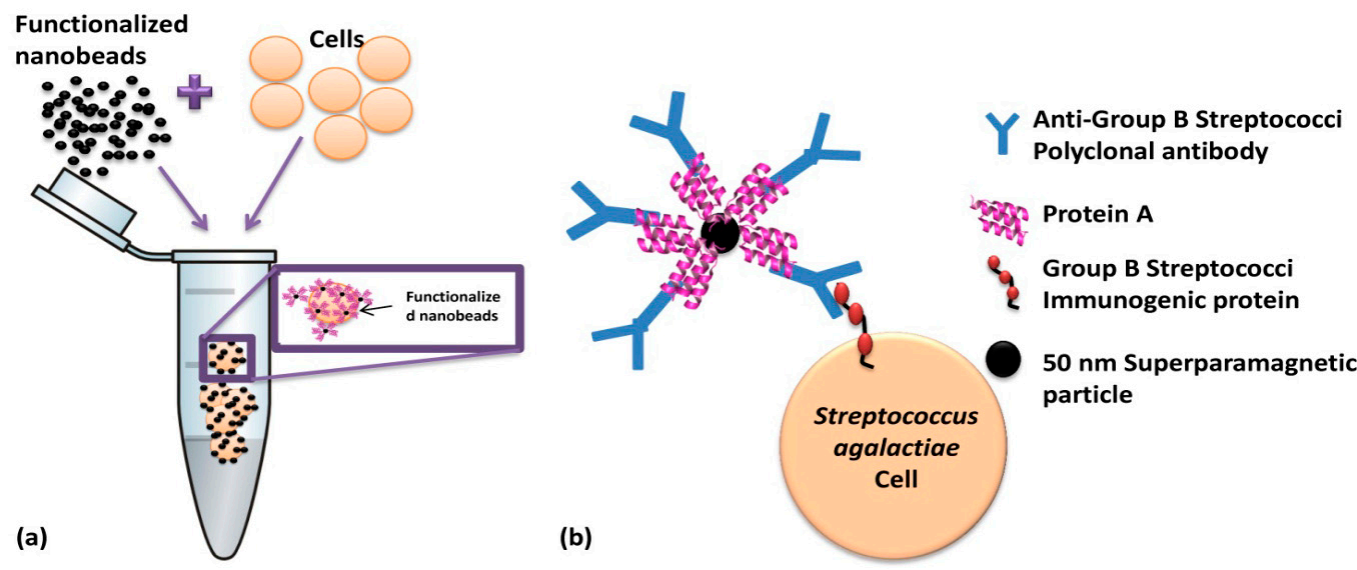
Schematics of immuno-magnetic functionalization of cells (**a**) Incubation of functionalized beads with *Streptococcus agalactiae* cells and (**b**) biological affinities between beads protein A, polyclonal IgG antibodies and bacterial cell wall epitopes.

In this work, magnetic particles are used as labels of polyclonal antibodies anti-Group B Streptococci (probes) which are going to recognize (via biomolecular recognition) bacterial *Streptococcus agalactiae* cells (targets) in the sample. Biological affinities between nanobead surface protein A, IgG Fc fraction and, the antibodies and Lancefield Group B Streptococci cell wall immunogenic proteins are illustrated in Figure 4. After labeling, the cells are introduced in a microfluidic channel and the SV sensor detects the fringe field of the magnetic labels bound around the target cell.

The sensors and microfluidic channels were microfabricated and optimized at INESC-MN for this application, using an acquisition setup adapted from a previous work [26]. The tests using raw milk in microchannels were carried out at INESC-MN. The cell culture and magnetic functionalization and labeling protocols were performed at CIISA, according to manufacturer protocols.

### 3.1. Beads Functionalization

Nanomag^®^-D-spio 50 nm particles (79-20-501, Micromod Partikeltechnologie GmbH, Rostock, Germany) were selected because they have protein A at surface and can bind up to five IgG. The calculation of beads number and the amount of pAb anti-Group B Streptococci (8435-2000 AbD Serotec, Kidlington, UK) was based on the *Streptococcus agalactiae* (CECT 183) concentration in samples and considering 400 times more beads than the estimation for cell surface area saturation (1600 particles/cell calculated based on bead and cell surface areas) with 50 nm nanoparticles. The nanoparticles number for cell surface saturation (1600) was estimated according to Wolfram Mathematica 7.0 calculations. A magnetic particles concentration of 6.4 × 10^9^ particles/μL was prepared for each sample in order to ensure the cells full coverage.

Particles were coated with 10.6 μL of pAb anti-Group B Streptococci (1 μg/mL) at room temperature (RT) incubation (25 °C), during 50 min. assisted with rolls plate agitation. Functionalized particles were magnetically separated by MS column (130-042-201 Miltenyi, Bergisch Gladbach, Germany) according to the MACS Miltenyi Biotec protocol.

### 3.2. Bacterial Cells Magnetic Labeling

*Streptococcus agalactiae* cells (CECT 183) were grown at 37 °C overnight on blood agar plates and resuspended in Tripticasein Soya Broth (TSB) over 24 h at 37 °C ([55] adapted protocol). After cell pellet collection through 2700 rpm centrifugation at 17 °C during 15 min. (HERMLE Z 383K centrifuge, Wehingen, Germany) and discarding the supernatant, PBS 1X (pH 7.2) buffer was added to absorbance reading at 600 nm (DU-68 Spectrophotometer, Beckman, Pasadena, CA, US) and for CFU/mL estimation. For incubation of 200 μL of magnetic particles with pAb anti-Group B Streptococci, milk and PBS volumes were prepared for final samples amount of 600 μL, and bacteria concentration of 10^4^ CFU/μL. Incubation was performed at RT for 50 min assisted with rolls plate agitation.

### 3.3. Milk Samples Preparation

Raw milk for experiments was collected aseptically from a healthy cow. As known, milk is a natural buffer with pH between 6.6 and 6.8 [56]. Conventional microbiological tests were performed accordingly with NMC (1999) [56] protocols, to confirm no bacterial growth. Briefly, a raw milk drop (10 μL loop) was smeared on a COS blood agar plate (43021, Biomerieux, Craponne, France) and a MacConkey agar plate (610028, Liofilchem Diagnostic, Roseto degli Abruzzi, Italy) made in the laboratory and both submitted to 37 °C during 48 h.

To achieve defatted milk samples, raw milk samples were frozen at −20 °C over 24 h and then unfrozen. During freezing, fat “cold agglutination” occurs forming a top layer of crystallized fat globules at milk surface. This layer was removed and milk underneath was used as “defatted” one.

### 3.4. Sensor Fabrication

#### 3.4.1. MR Sensor Fabrication

**Figure 5.**
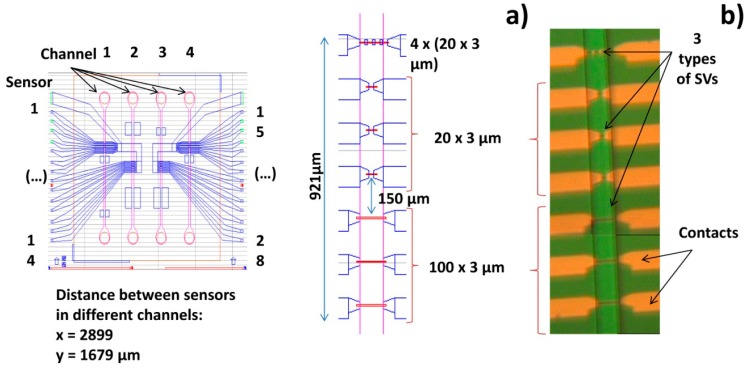
(**a**) Device CAD mask and sensor dimensions; (**b**) Microscope photo of one set of seven microfabricated SVs.

The chips fabricated in this work comprised 4 sets of rectangular SVs disposed in a line. Each SV set includes seven sensors with 3 μm width, and length varying from 20 to 100 μm (measured between contact leads), according with Figure 5. Sensor geometry was optimized to promote a linear, hysteresis-free transfer curve upon patterning into micrometric dimensions (sensor dimensions and shape definition). Additionally to individual sensors, we have included also four SV sensors connected in series. These configurations were designed to cover the width of the microchannel to be included above the chip. The sensors were fabricated on a 150 mm-diameter silicon wafer passivated with a 50 nm-thick Al_2_O_3_film deposited by Physical Vapor Deposition (PVD). The bottom-pinned SV thin film stack was deposited by Ion Beam Deposition (IBD) on a Nordiko 3000 device (Hampshire, UK) with the following structure (thickness in nm, compositions in atomic %): Ta 2.0/Ni_80_Fe_20_2.5/Co_80_Fe_20_2.3/Cu2.2/Co_80_Fe_20_3.3/Mn_76_Ir_24_7.0/Ta 10.0 [57]. During the deposition, a 3 mT magnetic field was applied in order to induce a parallel anisotropy simultaneously for the free layer (Ni_80_Fe_20_/Co_80_Fe_20_) and pinned layer (Co_80_Fe_20_) easy axis. Then, a 15 nm of Ti_10_W_90_ (N_2_) passivation layer was deposited by PVD in a Nordiko 7000 tool. SV definition was performed by direct write laser (DWL) lithography and ion milling in a Nordiko 3600 tool. The metallic contacts were defined by lithography and liftoff of a 300 nm-thick Al_98.5_Si_1.0_Cu_0.5_/15 nm-thickTi_10_W_90_ (N_2_) layer deposited by PVD in a Nordiko7000 tool. Deposition of 300 nm-thick Si_3_N_4_ passivation layer was carried out in Electrotech Delta Chemical Vapor Deposition System. Via definition was performed by lithography and reactive ion etching in LAM Rainbow Plasma Etcher 4400. After wafer microfabrication, dicing of the individual dies was done by a Disco DAD 321. Prior to sensor characterization, the wafer was submitted to a magnetic annealing at 250 °C for 15 min, in vacuum and cooled down under a 1 Tesla magnetic field.

#### 3.4.2. Microfluidic Channels Fabrication

**Figure 6.**
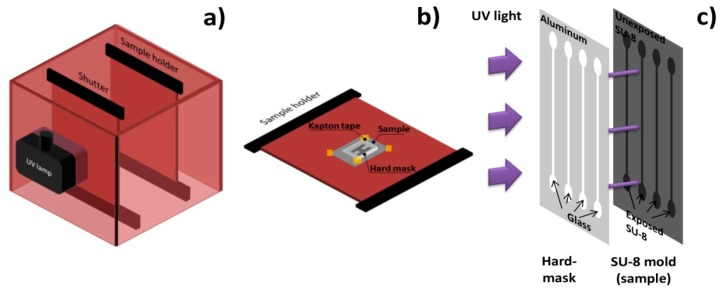
Schematics of the contact microlithography steps: (**a**) UV exposure setup; (**b**) Sample holder with hard-mask assembled over SU-8 substrate; (**c**) SU-8 negative resist exposure process.

A channel geometry of four single parallel channels with 100 μm height, 100 μm width and 1 cm length was considered. The inlets have a tear shape to ease cell entrance in the channels. Channels were made of poly (dimethylsiloxane) (PDMS) and fabricated by cast-molding, following a procedure similar to [58]. A hard-mask used to expose channels' mold was made of Al_98.5_Si_1.0_Cu_0.5_150 nm thick layer deposited on Corning glass by PVD in a Nordiko 7000 tool, patterned by DWL lithography and chemically etched with a solution of acetic acid (3.3%), nitric acid (3.1%) and phosphoric acid (3.0%). Channels' mold was fabricated by contact microlithography (Figure 6) of a 100 μm thick SU-8 50 photosensitive negative resist spun onto a silicon wafer and it was observed for defects in a DEKTAK 3030ST profilometer. In order to cast PDMS channels with a controlled thickness and shape and inlet/outlet aligned with microchannel, three 2 mm-thick poly (methylmethacrylate) (PMMA) plates were micromachined with a CNC TAIG Micro Mill tool. The resist mold was mounted on the bottom plate with Kapton tape, then the middle plate defined PDMS (2 mm) shape and thickness and the top plate defined inlet and outlet holes (1 mm). The two top plates possess alignment holes (2 mm).

#### 3.4.3. Bonding and Encapsulation

**Figure 7.**
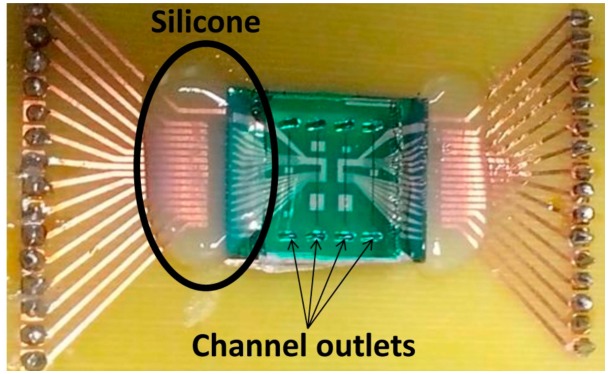
Wire-bonding silicon-protected assembled device.

Silicon chip integration with PDMS microchannels was achieved through irreversible bonding of the Si_3_N_4_ and PDMS surfaces by ultraviolet/ozone (UVO). Both surfaces were treated in a UVO Cleaner (Jelight, USA) for 15 min and immediately submerged in deionized water. Chip and microchannels were then mounted face-to-face, aligned on a microaligner with an *x*, *y* and theta direction stages under a microscope, using ethanol to delay bonding until complete alignment (Figure 5b). After drying at room temperature, the bonded device was placed in an oven at 70 °C for 30 min to complete irreversible bonding. Encapsulation was performed by mounting and gluing the bonded device on a Printed Circuit Board (PCB). The connection to the microfabricated sensors was done by wire-bonding the contact pads with the PCB. Finally, the wires were covered with silicone gel for protection. Figure 7 shows the final assembled device.

### 3.5. Samples Measurement

**Figure 8.**
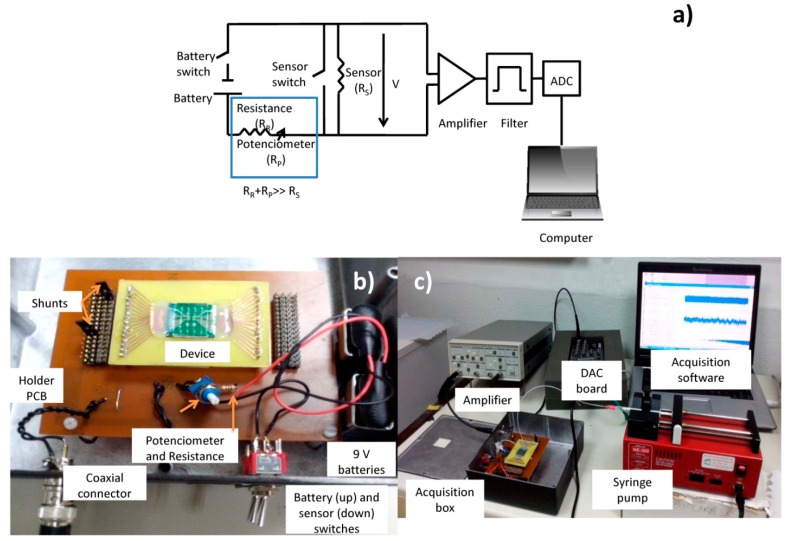
Acquisition Setup. (**a**) Electrical circuit of the acquisition setup; (**b**) Biasing box; (**c**) Acquisition setup assembly.

Sensor output signals were obtained using the electrical scheme shown in Figure 8. A 3 mA current was supplied to the sensor by two 9 V batteries in series (∼18 V), 1 kΩ resistance (R_R_), a potentiometer (R_pot_) set at 5 kΩ (R_R_ and R_pot_ together have a higher resistance than the sensor's average resistance, R_S_ ∼555 Ω). The output of the sensor was connected to acquisition setup composed by (a) an amplifier (Stanford Research Systems SR560, California, US) operating for gains of 10,000x, (b) high-pass and low-pass filters of 300 (to filter the DC and part of low frequency noise) and 10,000 Hz (to avoid aliaising), respectively and (c) a 16 bit analogue to digital converter (ADC) board DT9836-12-2-BNC (20 kHz acquisition frequency), which was connected to a laptop, where a home-made software was used to acquire sensor output *vs.* time (Figure 8b,c).

Each test required channel inlet sample introduction through capillary tubes (BTPE-90 polyethylene tubes, Instech Laboratories, Inc., Pennsylvania, PA, USA) plugged into a 1 mL syringe (Codan, Cat: 621640, LuerStubs LS20, Pennsylvania, PA, USA). Fluid flow was controlled by an automated syringe pump (NE-300 model, New Era Pump Systems, Inc., New York, NY, USA), and the sample was collected from the outlet by another capillary tube to a disposable Eppendorf.

## 4. Results and Discussion

In order to be able to understand experimental data, it is important to perform simulations of the signals varying the positioning of cells and relative positioning of cells inside the channel. It is also important to understand the variations of the signals for different directions of the magnetic moment.

### 4.1. Sensors Characterization and Magnet Calibration

**Figure 9.**
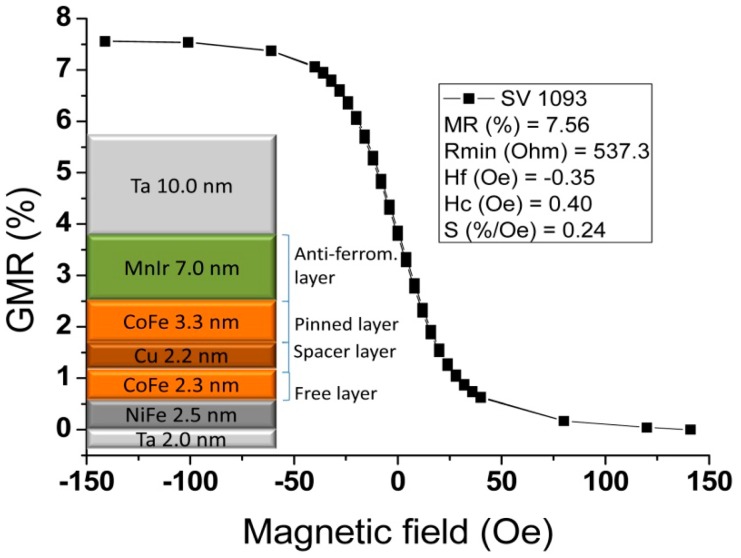
Transfer curve for a selected sensor with an area of 100 × 3 μm^2^. Inset shows the multilayer structure used for these sensors.

The sensors' transfer curve (resistance *versus* DC magnetic field up to 140 Oe) was characterized. Figure 9 shows a representative example of a 100 × 3 μm^2^ sensor, showing a linear range of 65 Oe, a sensitivity (S) of 0.24%/Oe, offset field (*H_coupling_*) of −0.35 Oe and coercivity (*H_c_*) of 0.40 Oe. A summary of the dispersion obtained over the 28 sensors measured on the chips fabricated is depicted in Table 1. From the table, one can confirm that connecting the sensors in series reduces the dispersion in all parameters because it averages the individual SV characteristics.

**Figure 10.**
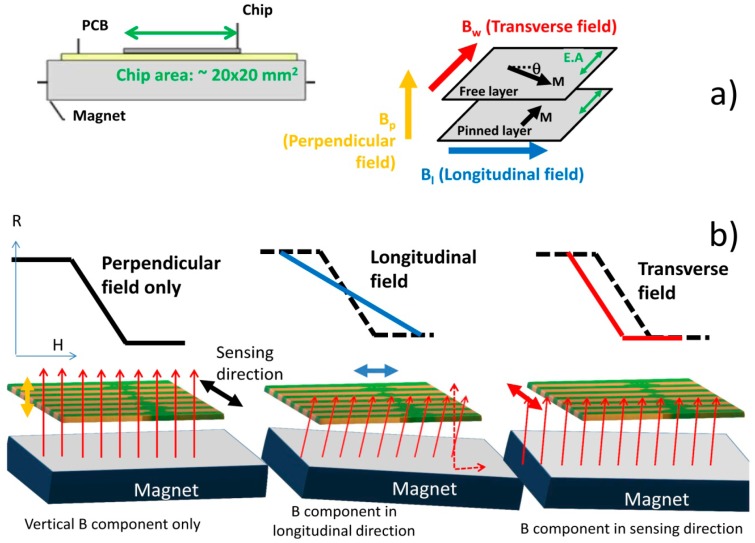
(**a**) Schematics of the geometry used for the nanoparticle magnetization using an external permanent magnet underneath the sensor and the magnetic field components (transverse, perpendicular and longitudinal) with respect to the free and pinned layer magnetization orientations; (**b**) Impact on the sensor response of each magnetic field component (set by magnet position) transfer curves.

To set a vertical magnetic field to magnetize the nanoparticles, a permanent magnet block (dimensions 20 × 20 × 3 mm^3^, NdFeB, Supermagnete, Gottmadingen, Germany) with ∼6.29 × 10^4^ A/m was mounted below the PCB with the bonded device. As the SV are only sensitive to an in-plane, if well aligned, the magnet will not affect the sensitivity of the sensor. However, a small tilting of the magnet can create magnetic field components in the sensor plane and therefore affect the sensor's sensitivity. The impact of the magnet positioning on the sensor transfer curve is illustrated in Figure 10. With a well aligned magnet, the sensor transfer curve is centered around zero external fields, with maximum sensitivity (Figure 10a). A slight tilt of the magnet creates fields in the longitudinal (Figure 10b) and/or sensing direction (Figure 10c) that shift the sensor transfer curve and/or decrease the sensor sensitivity, respectively. Therefore, combining magnet positioning and transfer curve measurements, it was possible to achieve a magnet position that did not degrade the sensor sensitivity (Figure 10a).

### 4.2. Sensors Magnetic Behavior, Signal Amplitude and Shape

In this work, several sensor dimensions were tested in order to ascertain the best configuration for dynamic single cell detection in a wide range of concentrations in terms of signal intensity and noise level (signal-to-noise ratio). Three different sensors were fabricated: four 20 × 3 μm^2^ sensors in series, a sensor with a large detection area (100 × 3 μm^2^) and a sensor with a small area (20 × 3 μm^2^). Both sensors in series and the large area sensor allow increasing the detection area available with a higher signal intensity, however are probably unable to distinguish between two or more cells flowing over the sensor simultaneously. In terms of magnetic labels, the chosen 50 nm superparamagnetic particles have a very low individual moment (∼2.7 × 10^−18^ Am^2^) and so cannot be individually detected, while having a magnetic moment sufficient for detection of a completely covered cell. Their small dimension reduces the probability of several cells to bind to a single particle causing cell aggregation.

#### Magnetic Detection Simulations

**Figure 11.**
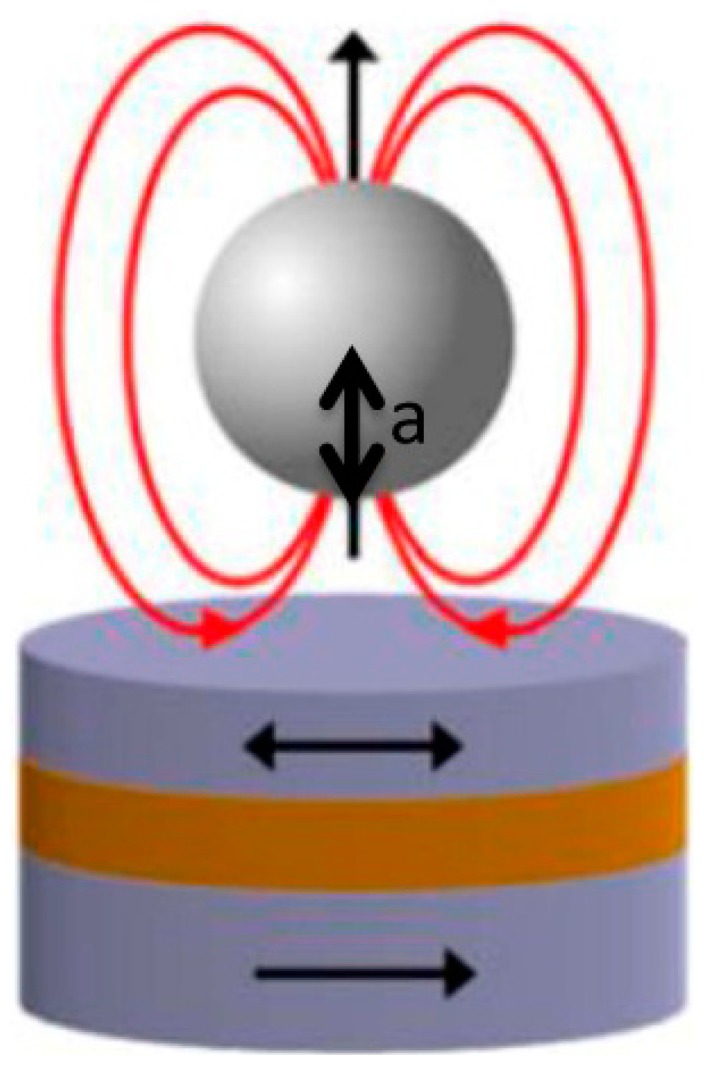
Out-of-plane magnetic field application for magnetic label sensing measurement schematics.

In order to assess the expected signal that would be obtained with each sensor dimension, a simulation of their sensing behavior and sensitivity to magnetic labeled cells was performed in Wolfram Mathematica 7.0. To simulate the magnetically labeled cells' behavior over sensor's free layer we considered that each particle labeling the cell could be approximated to a magnetic dipole centered on the geometrical center of the particle, which was considered a sphere (Figure 11).

Dipole's magnetic field created at the position *a* from dipole center is given by Equation (5):

(5) $$H(a)=\frac{1}{4\pi }\left(\frac{3\left(a\cdot m\right)a}{{\left|a\right|}^{5}}-\frac{m}{{\left|a\right|}^{3}}\right)$$

As SVs used in this work are only sensitive to transverse in-plane component of beads' fringe field, only the magnetic field parallel to sensor plane (*x*-component of fringe field) for particles with a magnetic moment (*m*) was calculated. The field below a particle at position (*x*, *y*, *a*) when it is magnetized perpendicularly to the sensor plane (*z* direction), is thus given by:

(6) $$H_{x}^{\text{perp}}\left(x,y,a\right)=\frac{m_{z}}{4\pi }\frac{3xa}{{\left(x^{2}+y^{2}+a^{2}\right)}^{5/2}}$$

**Figure 12.**
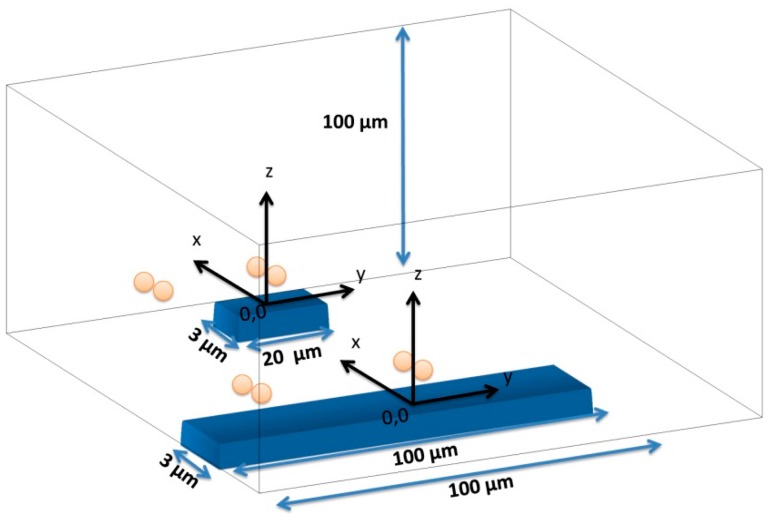
Schematics of sensor and channel geometry considered for output signal simulation in Wolfram Mathematica 7.0.

As SVs are sensitive to the average fringe field generated by magnetically labeled cell, $H_{x}^{\text{perp}}$ field of each particle has to be averaged over the sensor, which can be achieved by integrating Equation (6) over the sensor area and divide the result by the area of the sensor. In the specific case of this work, the fringe field of a cell (*H_cell_*) with 1 μm diameter fully covered with 50 nm superparamagnetic particles (1600 nanoparticles per cell) was calculated considering spherical cell and each particle occupying a circle area equivalent. Since each *Streptococcus agalactiae* cell is composed by pairs of 1 μm coccus, simulations were performed for pairs of spheres. The simulation was performed considering the geometry presented in Figure 12 with axis positioned in each sensors center and assuming sensors positioned in the center of the channel.

**Figure 13.**
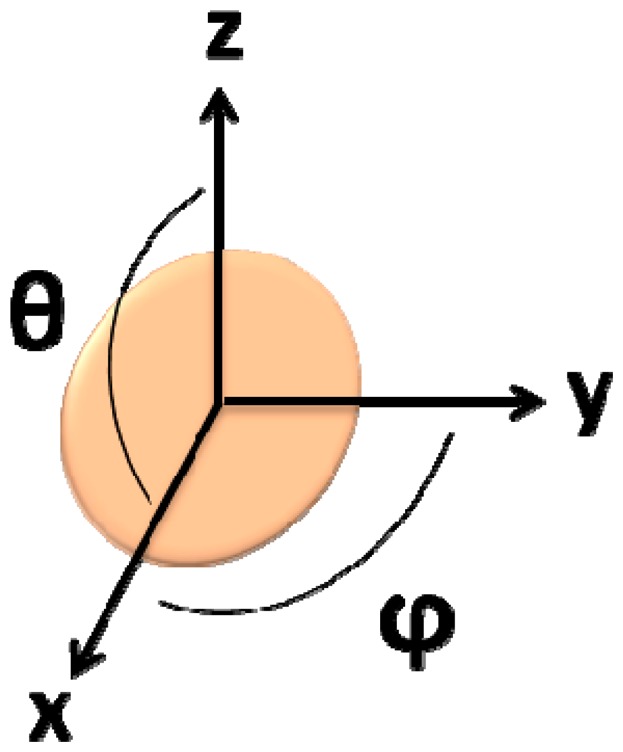
Axis and angles considered for Wolfram Mathematica 7.0 simulations.

**Figure 14.**
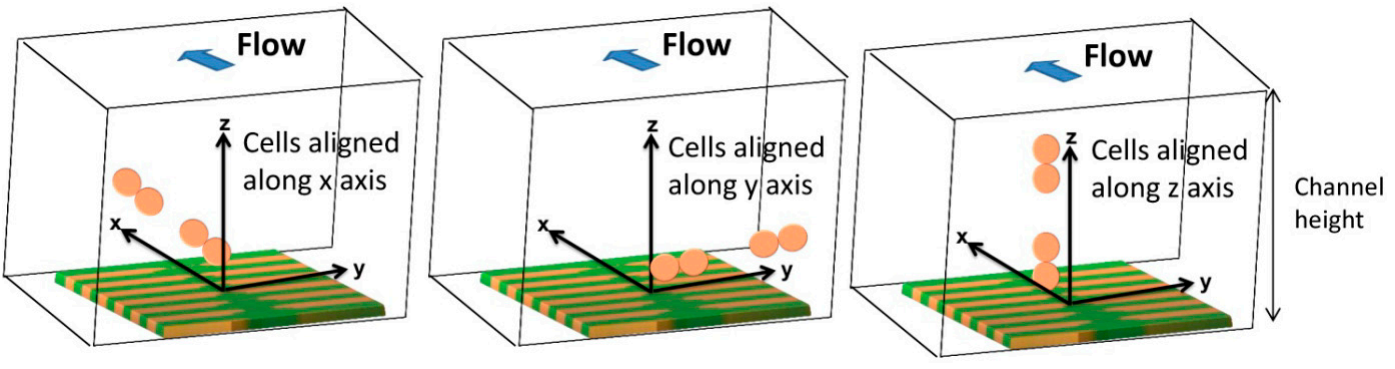
Schematics of cell orientations along *x*, *y* and *z* axis considered for cells in flow (*x* axis direction) in Wolfram Mathematica 7.0 simulations.

In this simulation, the fringe field generated by a single particle was calculated considering its position on the cell (Equation (7), where *c* is the cell's radius, *θ* is the angle within the *x*/*z* plane and ϕ is the angle within the *x*/*y* plane) (Figure 13), which was assumed as a pair of spheres flowing in the middle of the channel (Figure 14):

(7) $$\begin{array}{c} x=c\times \text{sin}\varphi \times \text{cos}\theta \\ y=c\times \text{sin}\varphi \times \text{sin}\theta \\ z=c\times \text{cos}\varphi \end{array}$$

Cells' fringe field was obtained for 62 different positions of *x* (0.8 μm apart covering a range from −25 μm and 25 μm in the *x* axis, considered the flow direction) and plotted against its corresponding position relative to sensor's center.

In order to obtain simulated signals similar to experimental ones, cells' fringe field was transformed into electrical signal (ΔV) using Equation (4), considering *H_ext_* = *H_cell_*, *H_bias_* = *H_coupling_* = 0, *I* = 3 mA and the sensors characteristics of Figure 14. Initially, signal simulations were performed considering one or two cell pairs oriented according to one of the axis (Figure 14) and at different height above the sensor.

**Figure 15.**
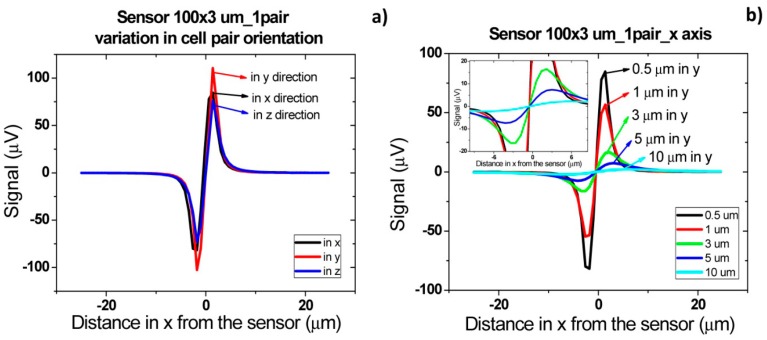
Signal simulation for *Streptococcus agalactiae*. (**a**) Pair of cells oriented according with each axis; (**b**) Signal variation with height from the sensor according with *x* axis.

In accord with simulations, different signal shapes and intensities were observed if the cell pair is oriented along *x*, *y* or *z* axis. In Figure 15a, when compared to the *x* and *z* orientation, an intensity increase of 200 μV was observed for y axis orientation. This is due to the fact that pair of cells was aligned along the length of the sensor and therefore the observed signal corresponds to two 1 μm cells. This is not the case for the other two orientations since in one case, the 1 μm cells are flowing one after the other (*x* orientation) and, in the other case, one of the 1 μm cells is more apart from the sensor leading to a signal attenuation. Nevertheless, these signals shape remain very similar and for simplification, only the pair of cells aligned in the *x* direction will be further analyzed. In Figure 15b, the signal variation with channel height for a cell pair is shown. It can be observed that the higher the cell flows along the channel the lower is the signal intensity. The amplitude of the bipolar peak above a height of 10 μm is below 15 μV. Since the sensor noise is 10–15 μV [30], we can conclude that cells passing at a height above 10 μm are unable to be detected.

Besides the specific cells presence detection, the final goal of this device will be also to set known cells concentration in samples. It is therefore important to understand which signal shapes are obtained when two pairs of cells are flowing one after the other, side by side or one on top of the other.

**Figure 16.**
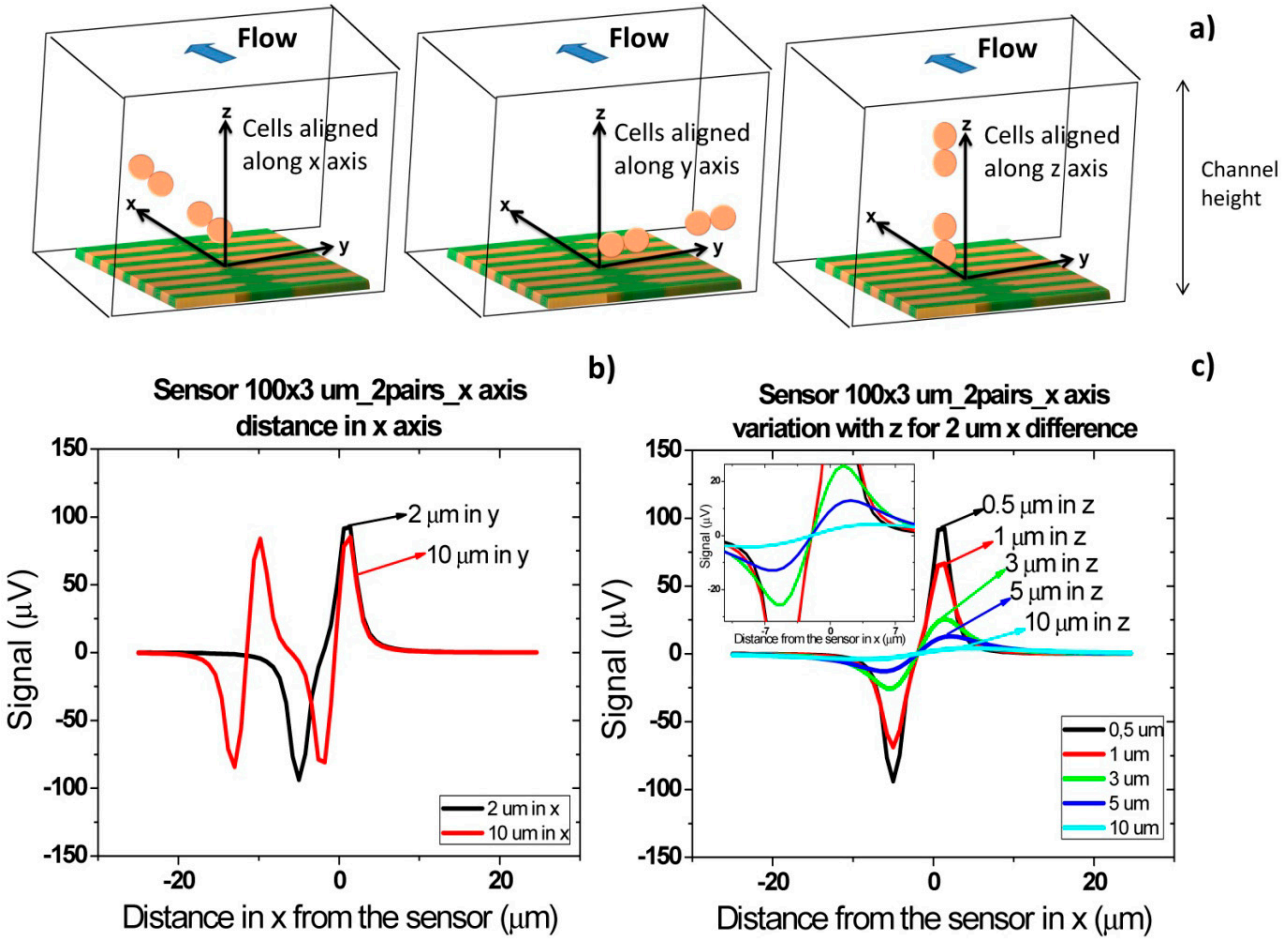
Signal simulation for two *Streptococcus agalactiae* along *x* axis. (**a**) Schematics of cell pair's orientation considered for simulation; (**b**) Cells at a given distance along *x* axis; (**c**) Signal variation with height from the sensor for cells at 10 μm distance from each other along *x* axis.

Figure 16b shows the signal of two pairs of cells flowing one after the other over the sensor with a separation of 2 and 10 μm. For a cell pair distance of 2 μm, a bipolar signal is observed with a slight slope variation near zero and no variation in the amplitude when compared to a single pair of cells. For a distance of above 10 μm between each pair, the signal of each cell is already decoupled and two bipolar peaks are observed. These signatures permit the identification of two pairs of cells passage one after the other. Furthermore, by increasing the height of the cells, as expected, the signal amplitude is reduced (Figure 16c). As discussed previously, for heights above 10 μm, the signal is below the expected sensor noise and therefore the sensors are unable to detect it.

**Figure 17.**
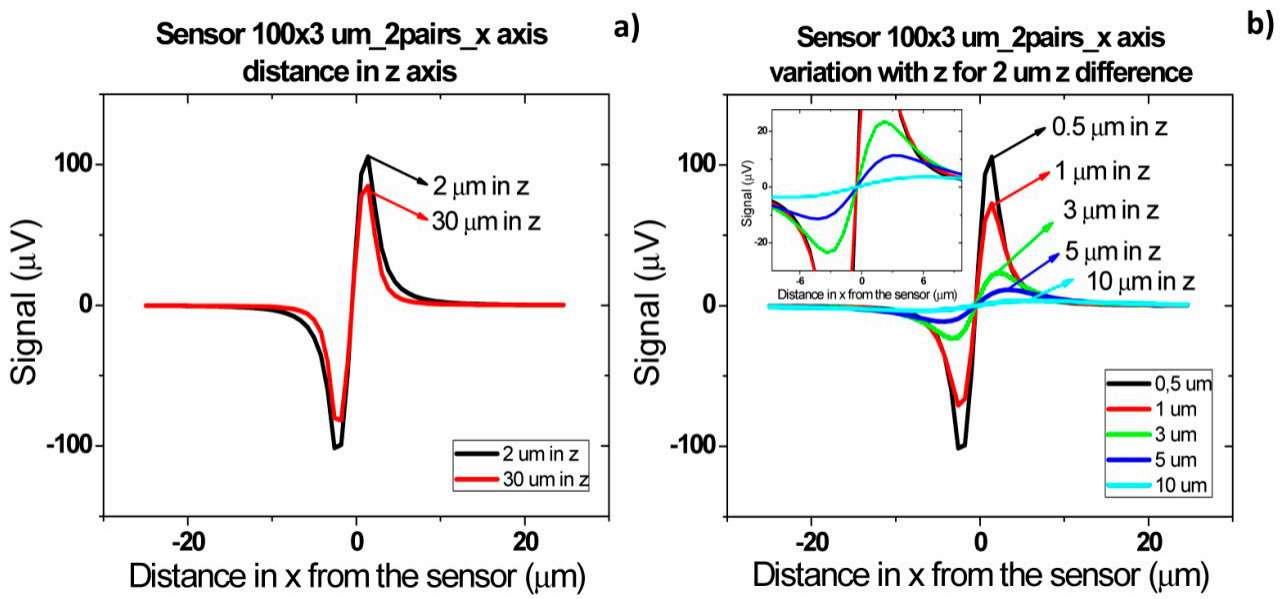
Signal simulation for two *Streptococcus agalactiae* along *z* axis. (**a**) Cells at a given distance along *z* axis; (**b**) Signal variation with height from the sensor for cells at 2 μm distance from each other along *z* axis.

For cells flowing simultaneously above the sensor at different heights (Figure 17) similar peaks are observed. As can be observed in Figure 17a, for separations larger than 30 μm, there is an attenuation of 200 μV. This is due to the fact that in this case only the cell closer to the sensor generates a fringe field high enough to be detectable. Again, as expected, when the two pairs of cells (2 μm separated from each other) flow at a height above 10 μm, the signal is within the sensor's noise enabling it to detect the cells.

**Figure 18.**
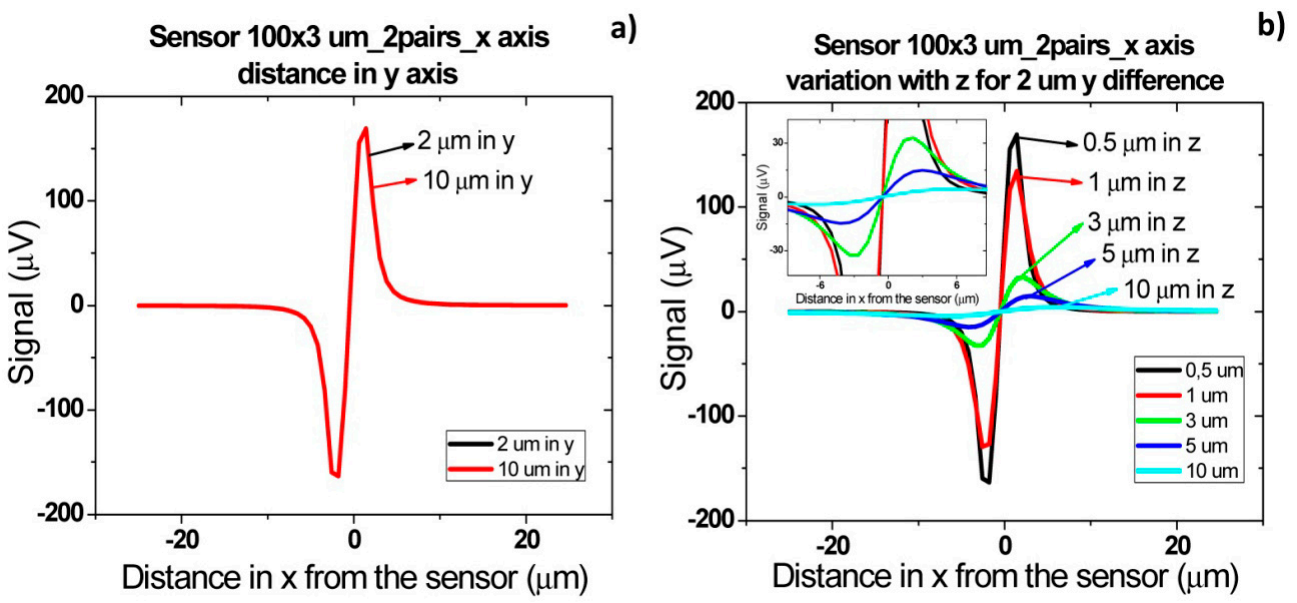
Signal simulation for two *Streptococcus agalactiae* along *y* axis. (**a**) Cells at a given distance along *y* axis; (**b**) Signal variation with height from the sensor for cells at 10 μm distance from each other along *y* axis.

Finally, when cells flow simultaneously through sensor at different positions in *y* direction (Figure 18), a signal increase corresponding to sum of each individual cell signal (Figure 18a) is observed. As sensor is 100 μm long, which corresponds to channel's width, the signal will remain independent of cells separation distance in y direction. As in previous cases, the amplitude of bipolar peaks will be attenuated as cells flow more apart from sensors.

**Figure 19.**
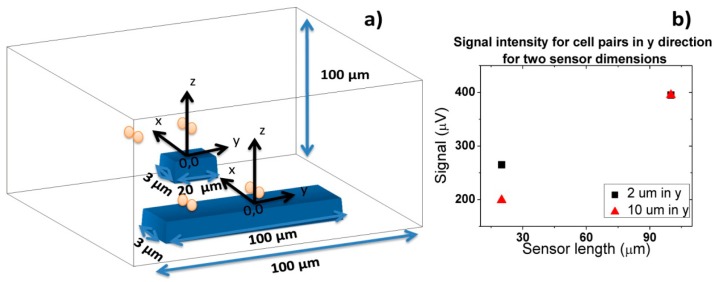
(**a**) Schematics of cell flow over two types of SVs; (**b**) Signal amplitude for two types of SVs obtained from simulations for two pairs of cells oriented along *y* axis and separated by 2 and 10 μm.

Sometimes, in real data it is not easy to distinguish if two cells are flowing side by side in *y* direction, since a large signal can correspond to several cells flowing simultaneously at a specific height or to a single cell pair flowing at a lower height. This fact would lead to an underestimation of detected cells number. To avoid this, one can spread several small sensors over the channel's width and measure them in parallel. Assuming that one of these sensors would have an area of 20 × 3 μm^2^, simulations were carried out for two pairs of cells separated by 2 and 10 μm in *y* axis. As expected from Equation (4), a lower signal is obtained for these sensors than for 100 × 3 μm^2^ sensor in the same conditions (Figure 19). Plus, due to sensor's smaller length than the channel, there is a decrease in signal detection outside sensor area due to dilution of cell magnetic fringe field with distance. Thus, cell pairs flowing outside 20 × 3 μm^2^ sensor's sensitive/detection will not be observed. Spreading the small sensors would therefore give more reliable results than using a single sensor occupying the channel's width. However, from the electronics point of view, a more complicated system would be required, to allow acquisition of several sensors in parallel.

**Figure 20.**
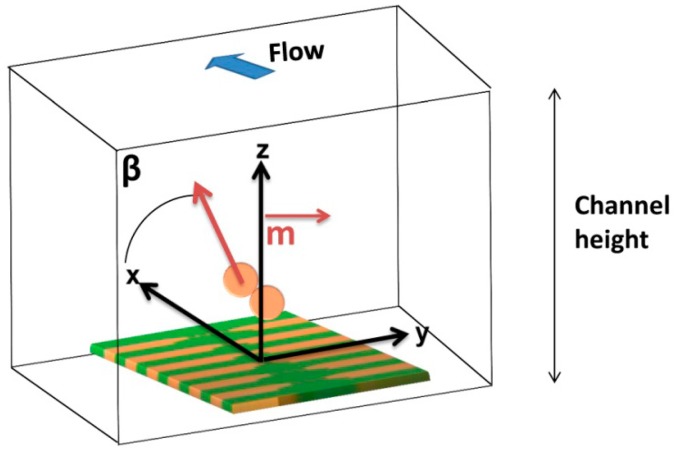
Schematics of axis and configuration considered for simulation in Mathematica 7.0 of the influence of both perpendicular and parallel components of fringe field in signal.

Finally, it has previously been observed that cell rotation due to flow or non-vertical magnetization direction influences signal peak shape observed [26]. Thus, the influence of a magnetic moment component in the *x* direction (Figure 20) was analyzed by introducing Equations (9) and (10) in the simulation:

(8) $$H_{x}^{\text{tot}}=\frac{m_{z}}{4\pi }\frac{3xa}{{\left(x^{2}+y^{2}+a^{2}\right)}^{5/2}}-\frac{m_{x}}{4\pi }\left(\frac{3x^{2}}{{\left(x^{2}+y^{2}+a^{2}\right)}^{5/2}}-\frac{1}{{\left(x^{2}+y^{2}+a^{2}\right)}^{3/2}}\right)$$

(9) $$\begin{array}{c} m_{z}=m_{s}\times \text{sin}\beta \\ m_{x}=m_{s}\times \text{cos}\beta \end{array}$$

where *m_s_* is the saturation magnetic moment of the particles and for the 50 nm particles used in this work *m_s_* = 2.7×10^−18^ Am^2^.

**Figure 21.**
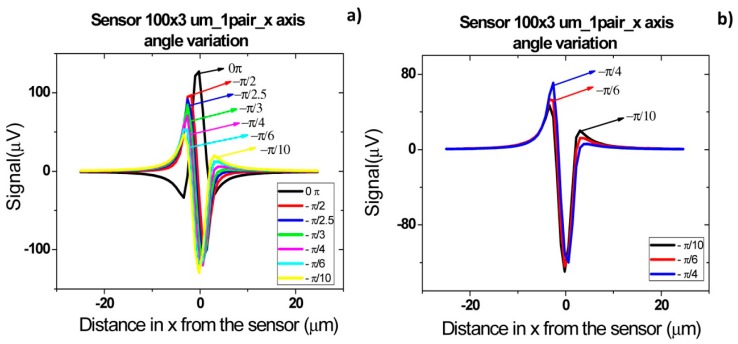
Signal simulation for detection of a *Streptococcus agalactiae* cell using a 100 × 3 μm^2^ sensor (**a**) oriented along *x* axis considering different magnetic moment angles relative to the *x* axis; (**b**) Selection of simulated angles.

Figure 21 presents signal variation with angle β between perpendicular and parallel magnetization directions. It is possible to observe that the introduction of parallel component of fringe field is translated as an asymmetric bipolar signal, which intensity decreases with higher β, since the contribution of perpendicular component decreases. When the magnetization direction is parallel to *x* axis a unipolar peak is observed.

In summary, by analyzing peaks shape and amplitude, it is possible to determine the orientation of magnetic moment and the number of cells as well as cell's height when flowing over the sensor.

### 4.3. Detection of Streptococcus Agalactiae Cells

For a first experiment, three samples were considered:

(i) raw milk as collected;
(ii) milk spiked with 200 μL of functionalized nanoparticles (6.4 × 10^9^ particles/μL for 10^4^ cells/μL detection) (after MACS separation of buffer solution with 145 μL of magnetic nanoparticles and 10.6 μL of pAb anti-GB Streptococci, per sample);
(iii) Similar to (ii) and also spiked with 4.6 μL of *Streptococcus agalactiae* cells suspension (1.28 × 10^6^ cells/μL). The samples were first incubated for 50 min and injected inside the microfluidic channel at a 50 μL/min flow rate. Results presented were obtained from repeated trials (three times for each sample) and with at least ten measurements for each flow rate.

**Figure 22.**
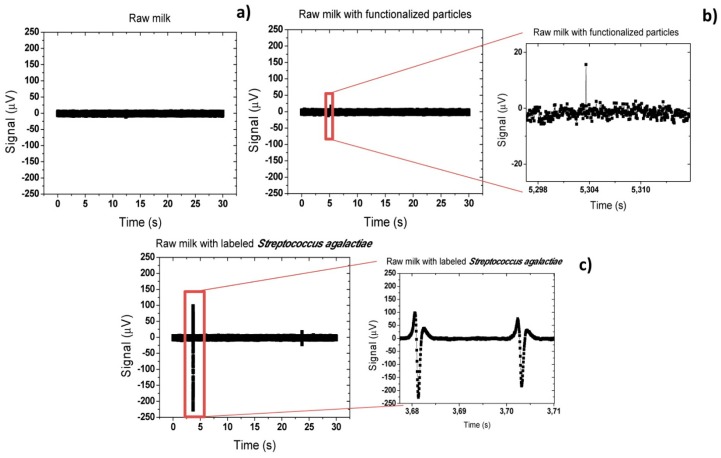
Acquired signal for raw milk samples (**a**) as collected; (**b**) spiked with functionalized nanoparticles and (**c**) spiked with magnetically labeled *Streptococcus agalactiae* cells at 50 μL/min*.*

Figure 22a shows that for the sample (i), no peak was observed and only the background noise of the sensor was observed. A peak-to-peak noise of 20 μV was measured. On the other hand, on sample (ii), where only functionalized magnetic particles were mixed with the raw milk, sporadically small peaks (<40 μV) appeared (Figure 22b). This may be explained by formation of small particles agglomerates due to applied magnetic field or due to adsorption of particles to milk constituents. From this point, all peaks above 40 μV of peak-to-peak amplitude were considered as positive bacterium detection peak. In fact, on samples (iii) including magnetically labeled S*treptococcus agalactiae* cells, large peaks (∼ 325 μV) were observed proving a positive detection of cells. As can be observed in Figure 22c, the measured peaks were unipolar. This indicates that magnetic moment of magnetic particles was almost oriented in the *x* direction (β ∼ π/10). This seems contradictory since a vertical magnetic field is applied during the experiment. In fact, this phenomenon was already observed in the past for magnetic particles [26] in a similar system and was associated to rolling of the magnetic particles over the sensor surface. However, this was the first time that such behavior was observed in cells.

**Figure 23.**
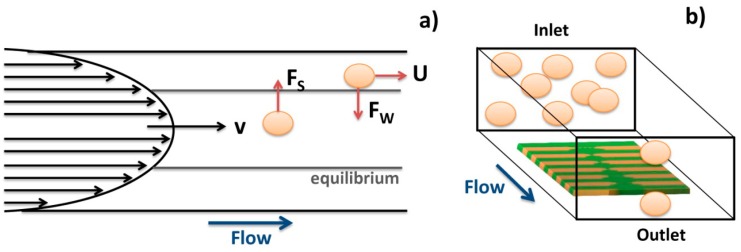
Schematics of inertial focusing in square microchannels. (**a**) Two lift forces in action, wall-induced *F_W_* and shear-induced *F_S_* lift forces; (**b**) In square channels randomly distributed particles or cells focus into four equilibrium positions at the wall centers.

Analyzing the literature, several works reported that in square or rectangular channels cells tend to focus on four equilibrium regions centered at channel edge as shown in Figure 23 [59,60].

This is valid for dilute suspensions of particles or cells flowing at moderate Reynolds numbers [59]. Considering that in this work, cells are magnetically label and the magnet is positioned bellow sensors, there may be a magnetic force pushing cells downwards. This small magnetic force associated to the equilibrium regions will therefore pull cells towards sensor surface. The surface drag force associated to the parabolic liquid velocity will further cause rolling of cells over the sensor.

**Figure 24.**
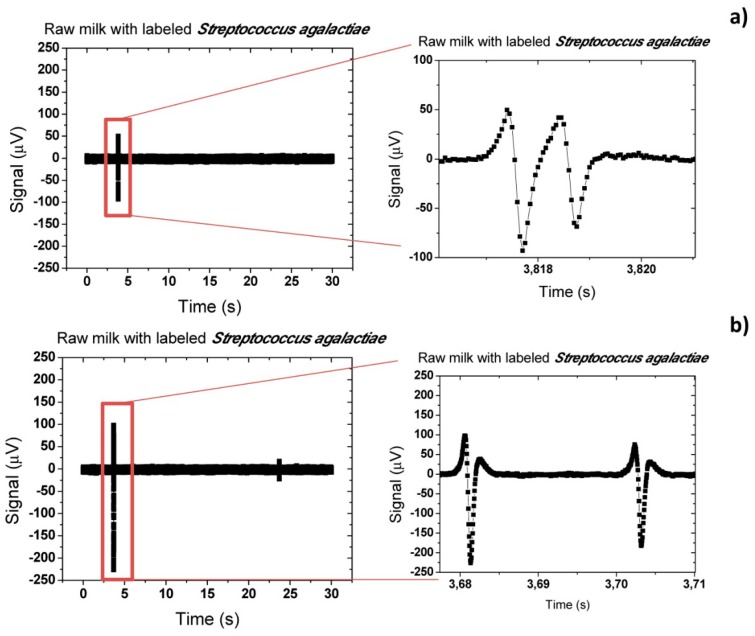
Acquisition signal for raw milk samples with magnetically labeled S*treptococcus agalactiae* cells at 50 μL/min*.*

On other trials, more symmetric peaks were observed (Figure 24) and with a lower amplitude. This demonstrates that in some cases cells flow above the sensor without rolling over the surface. When compared to the simulations of Section 4.1 (Figure 15b), peaks amplitude of 111 μV and 143 μV indicate cells positioning of 3–5 μm above sensor. Furthermore, peaks observed in Figure 24a are superimposed showing that at least two cells are flowing very close to each other but still far enough to distinguish two bipolar peaks. Comparing to simulations (Figure 16b) this indicates that cells are flowing one after the other at a separation ranging from 5 to 10 μm. This fact may be explained by some cell agglomeration at the inlet and consequent sporadic magnetic labeled cells release, generating peaks with these characteristics.

Based on the peak analysis, it is possible to estimate the concentration of bacteria in solution. Assuming an average of four peaks per acquisition of 30 s, a flow rate of 50 μL/min (meaning 25 μL of sample volume) and that each peak corresponds to a single cell, a bacteria concentration of 0.14 cells/μL is obtained. This value is far below the concentration of the input sample (10^4^ CFU/μL). This fact reinforces the assumption that there is a strong agglomeration of cells at channel's inlet and that only few cells are released into the channel and measured. This agglomeration could be explained by the large vertical (*z* direction) magnetic gradients created by magnet at channel's inlet. Some cells could only be released by the Stoke force created by a large flow rate (50 μL/min). Therefore, further optimizations of the system could include new inlet geometry, more homogeneous external magnetic field and/or higher flow rates, aiming at reduction of magnetic agglomeration at the inlet.

The platform's quantitative detection limit still requires further testing, however a limit of 10 CFU/μL has already been attained in a yes/no answer format as described in our subsequent work [61].

**Figure 25.**
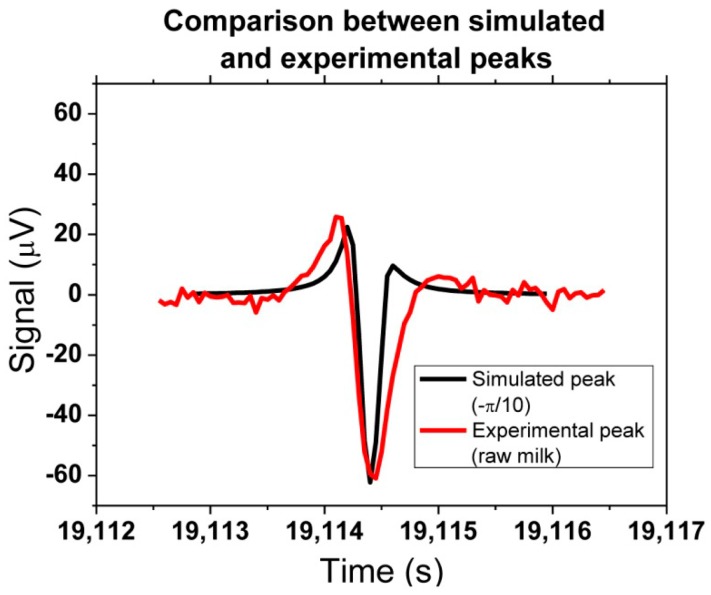
Direct comparison of experimental and simulated peaks. The simulation considered two S*treptococcus agalactiae* cells flowing at 50 μL/min, with a rotation of the magnetization by an angle of −π/10 with respect to the vertical.

A quantitative output of the platform can be obtained, through correlation between experimental and simulated peaks. One example can be seen in Figure 25, where one experimental peak from raw milk with bacteria could be compared with the simulation assuming a −π/10 rotation (in *x* direction). The amplitude of 60 μV was achieved by considering that two agalactiae cells were contributing to the detected signal.

**Figure 26.**
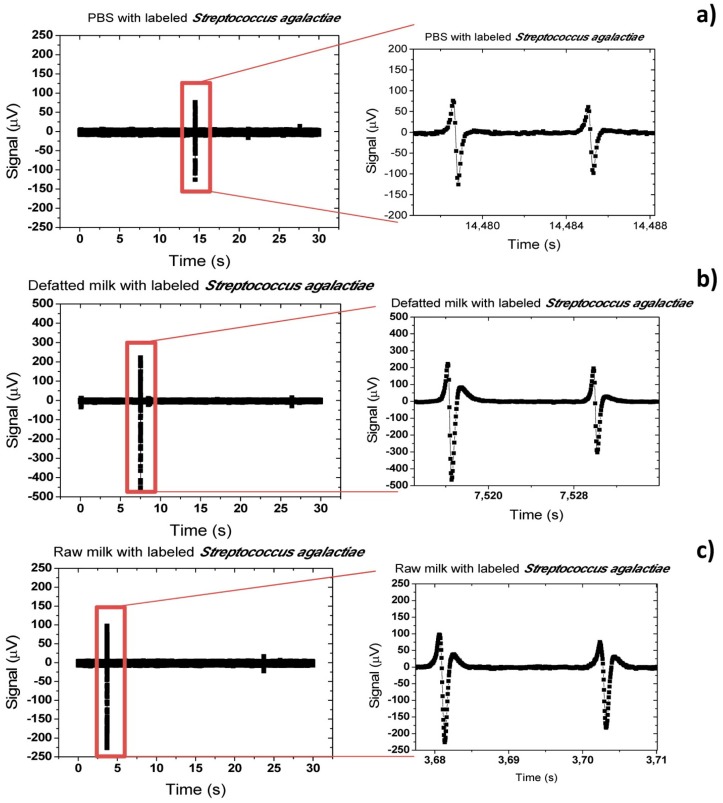
Acquisition signal of (**a**) PBS; (**b**) defatted and (**c**) raw milk samples spiked with magnetically labeled S*treptococcus agalactiae* cells at 50 μL/min.

Finally, a study comparing measurements in raw milk and defatted milk samples was performed. As observed in Figure 26b,c, the peaks' shape in defatted and raw milk are very similar and, as discussed previously, indicate that cells are rolling over sensor's surface. However, peaks in defatted milk show almost twice the amplitude than peaks in raw milk. This can be explained by the fact that raw milk is a more complex solution than defatted milk and therefore its constituents (fat globules, casein, *etc.* can be blocking the binding of magnetic nanoparticles to bacteria. This fact leads to a lower load of magnetic nanoparticles on bacteria inside raw milk and thus lower signals are measured.

On the other hand, PBS solution presents smaller amplitude peaks than milk samples (Figure 26a). Meaning probably that bacterial cells have less magnetic particles bonded. It is coherent with its simpler saline composition, unlike milk complexity, explaining more individualized bacteria cells. However, milk samples larger peaks show us that sensor might detect bacteria cells agglomerations due to favorable immunological bonding conditions existing in milk.

## 5. Conclusions/Outlook

This work describes a platform for in-flow detection of magnetically labelled cells with a magnetoresistive based cell cytometer, as an inexpensive and portable alternative to current flow cytometry in bio-applications. Sensor output response to cells and particles were simulated to obtain the impact of their position in the microchannel with respect to the sensor. Simulations indicated that the analysis of the different peaks' amplitudes and shapes can infer the position of the cells inside the channel and eventual simultaneous passage of two cells over the sensor. Experiments performed in raw milk, defatted milk and PBS buffer demonstrated specific detection of *Streptococcus agalactiae* cells. The results indicate that raw milk constituents (fat globules, casein, *etc.*) inhibit the bonding of nanoparticles to the bacteria leading to lower signal amplitudes. On the other hand, quantification is still an output to be improved since the cells seem to agglomerate at channel's inlet due to a strong magnetic gradient. To overcome this limitation, a different inlet/channel design, more homogeneous external magnetic field and/or higher flow rates could reduce this cells agglomeration deliver more reliable quantification values.

## Figures and Tables

**Table 1.** Results of SVs transport characteristics [28 sensors measured].

Sensor Dimensions [μm^2^]	MR (%)	R_min_ (Ω)	H_f_ (Oe)	H_c_ (Oe)	S (%/Oe)
**100 × 3**	7.59 ± 0.28	554.61 ± 20.65	1.01 ± 2.07	0.81 ± 0.74	−0.24 ± 0.03
**20 × 3**	6.58 ± 1.74	179.05 ± 39.31	−1.74 ± 2.98	2.79 ± 3.01	−0.19 ± 0.09
**In series**	7.51 ± 0.11	608.83 ± 4.79	−3.59 ± 0.47	2.40 ± 0.34	−0.18 ± 0.04
